# Double giant ectasia of right coronary artery in a young woman: A rare case report

**DOI:** 10.1016/j.ijscr.2020.04.099

**Published:** 2020-05-21

**Authors:** Tran Quyet Tien

**Affiliations:** a Department of Cardiovascular and Thoracic Surgery, Faculty of Medicine, University of Medicine and Pharmacy at Ho Chi Minh City, 217 Hong Bang Street, District 5, Ho Chi Minh City 72714, Viet Nam; b Cardiovascular Center of Cho Ray Hospital, 201B Nguyen Chi Thanh Street, District 5, Ho Chi Minh City 72714, Viet Nam

**Keywords:** CAE, coronary artery ectasia, CAA, coronary artery aneurysms, RCA, right coronary artery, Coronary artery ectasia, Coronary artery aneurysm, Giant

## Abstract

**Background:**

Coronary artery ectasia (CAE) is a rare condition that is characterized by the excessive diffuse dilatation of the coronary arteries by 1.5 times in diameter compared to adjacent coronary arteries. The giant CAE is even rarer and life-threatening.

**Case presentation:**

A 34-year-old female patient was admitted to our centre with three months history of vague chest pain that worsened gradually. The CT scanner confirmed that there were two giant aneurysmal sacs in the right coronary artery (RCA). The first sac was 32 × 36 mm in size and located in the proximal RCA, the second one containing thrombus in the middle RCA with a size of 43 × 30 mm. The patient was successfully treated by aneurysmal resection and concomitant coronary artery bypass.

**Conclusion:**

Giant coronary artery ectasia is a rare clinical condition and very dangerous for patients unless properly treated. We successfully treated a case of giant right coronary artery ectasia with favorable outcome.

## Highlights

• Giant coronary ectasia is a rare and life-threatening condition.
• The optimal strategy for the treatment of coronary artery ectasia is debatable.
• Surgical treatment is a proper method for the giant aneurysmal sacs.

## 1 Introduction

Coronary artery ectasia (CAE) is a rare condition that is characterized by the excessive diffuse dilatation of the coronary arteries by 1.5 times in diameter compared to adjacent coronary arteries [1,2]. Approximately, it only accounts for approximately 0.3–4.9% of cases detected on coronary angiography. The incidence in men is more prominent than in women (2.2% versus 0.5%, respectively) [1,3]. The CAE with diameter greater than 5 mm is even rarer with the prevalence of 0.02% and has only been sporadically reported in global literature [[4], [5], [6]]. The causes of this condition include atherosclerosis (50%), congenital malformations and Kawasaki disease (17%), infection and mycotic lesions (11%), connective tissue disorders and Marfan's syndrome (<10%) and iatrogenic complications (rare) [5]. Unless properly treated, this abnormality can cause life-threatening complications such as myocardial infarction or aneurysmal rupture [[7], [8], [9]]. Until now, there is no consensus in the management of CAE because of the lack of randomized trials and the rarity of this desease. Herein, we report a case of giant CAE involving the right coronary artery in a young woman who was successfully treated by aneurysmal resection and coronary artery bypass. The patient provided written informed consent, this report was approved by institutional review board, and it has been reported in line with the SCARE criteria [10].

## 2 Presentation of case

**Fig. 1:**
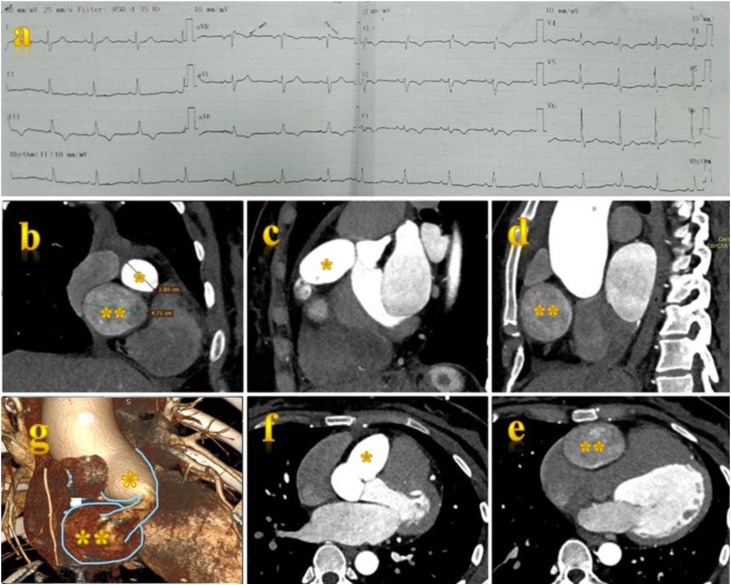
The CT scanner detected two giant aneurysmal sacs on the right coronary artery. (a) the 12-lead electrocardiogram showed sinus rhythm with a rate of 70 beats and incomplete right bundle branch block; (b) Coronal plain view of CTA; (c,d) Sagital plain view of CTA; (e,f) axial plain view of CTA; (g) three-dimension view of aneurymal sacs; (*) the first aneurymal sac; (**) the second aneurymal sac.

A 34-year-old female patient was admitted to our centre with three months history of vague chest pain that worsened gradually. She had an unremarkable medical history without Kawasaki disease or chest trauma. On initial physical examination, the vital signs were normal, blood pressure was 110/70 mmHg, heart rate of 76 beats per minute and peripheral capillary oxygen saturation of 97%, body mass index was 22. On diagnostic work up, the 12-lead electrocardiogram showed sinus rhythm with a rate of 70 beats and incomplete right bundle branch block (Fig. 1a). All hematological, biochemical, cardiac enzymes and immunological tests (hepatitis markers, HIV, Antinuclear antibodies – ANA) were unremarkable. However, the transthoracic echocardiogram unexpectedly detected a huge cardiac mass causing compression of the right atrium, right ventricle, tricuspid valve. This mass was hypoechoic echogenicity and had swirling flow suggesting the diagnosis of an aneurysm sac. Left ventricular systolic function was normal (63%).

The chest computed tomography (CT) scanner was immediately indicated and it confirmed that there were two giant aneurysmal sacs in the right coronary artery (RCA) (Fig. 1g). The first aneurysm was 32 × 36 mm in size and located in the proximal RCA, the second one containing thrombus was 43 × 30 mm in the middle RCA (Fig. 1b,c,d,e,f). The segment of artery between the first and second aneurysm was smaller measuring 8 mm in maximal diameter. The total length of the dilated coronary artery was 86 mm. Two aneurysms compessed the right atrium and ventricle. CT did not detect any fistula between coronary system and cardiac chamber or great vessel.

**Fig. 2:**
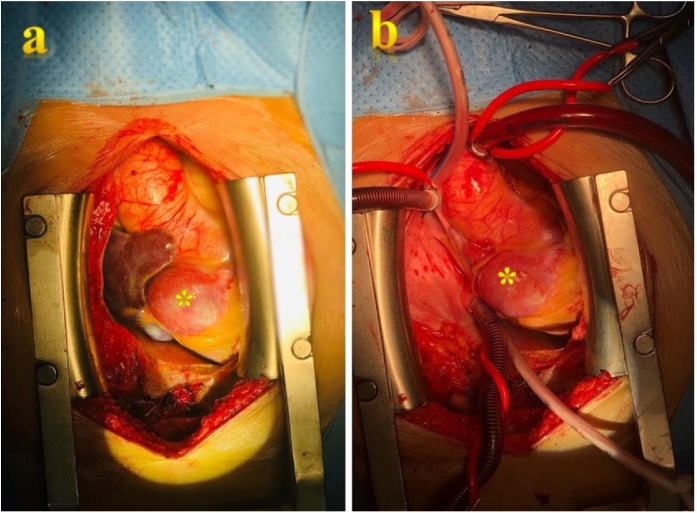
(a) The intraperative image of proximal aneurysmal sac (*). (b) The cardiopulmonary bypass was set through the ascending aorta, superior and inferior vena cava.

**Fig. 3:**
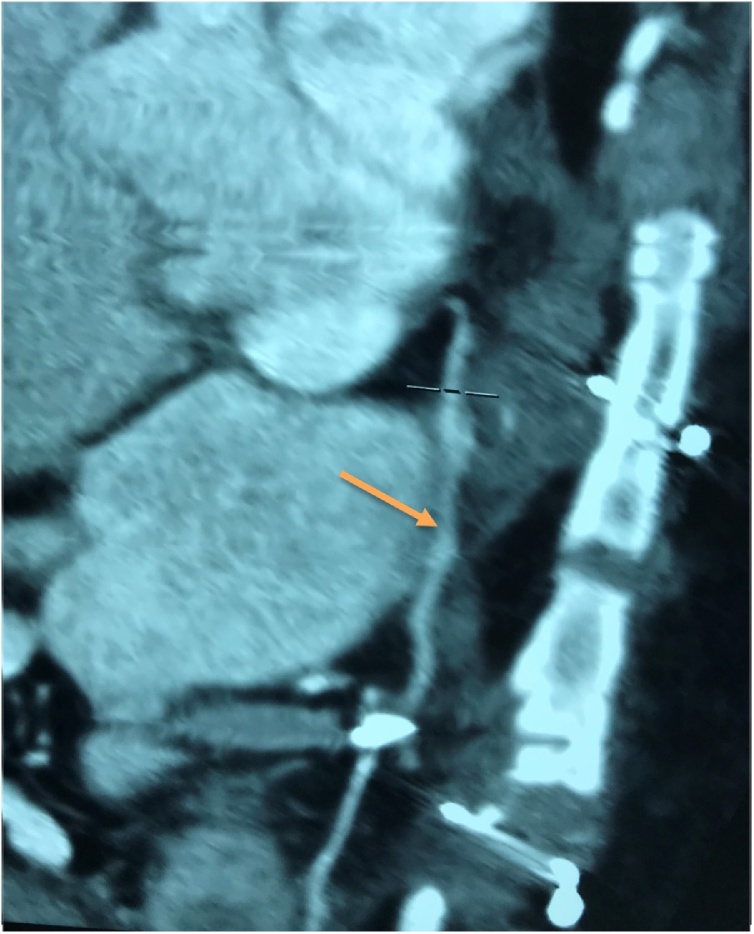
The postoperative enhanced CT of the chest was performed one-month post-CABG and the coronary reconstructions by GSV revealed patent (orange arrow).

**Fig. 4:**
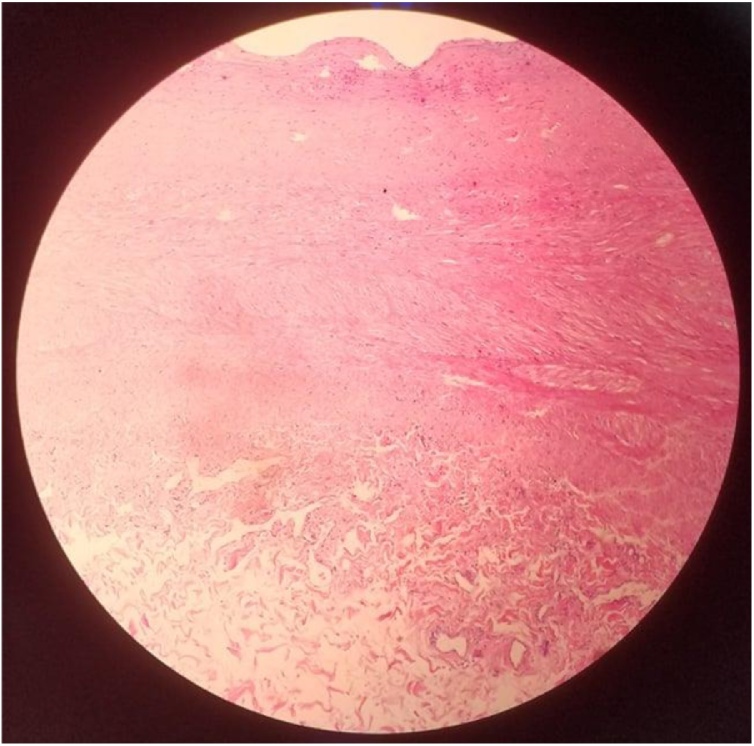
Histopathological examination showed the degeneration due to atherosclerosis.

The patient was in life-threatening situation because of the high risk of aneurysmal rupture, so she was immediately transferred to Cardiovascular Center. An urgent multidisciplinary consultation, including anaesthesiologists, cardiovascular surgeons, intensivists, decided to perform aneurysmal ligation, resection and coronary artery bypass graft. Under general anesthesia, the urgent procedure was carried out via full median sternotomy with the support of cardiopulmonary bypass (CBP) with hypothermia at 32 °C. CBP was instituted using aorto bicaval cannulation. Heart arrest was achieved with both Custodiol retrograde cardioplegia and antegrade cardioplegia. Two aneurysmal sacs (Fig. 2a,b) with thrombus were exposed, until approach the normal distal and proximal coronary arteries diameter, inflow ligated and resected, the outflow was sutured. Partial resection of the aneurysmal sacs was also performed. Coronary artery bypass grafting (CABG) was performed using a segment of the great saphenous vein (GSV) anastomosed to the distal right coronary artery distally and ascending aorta proximally. Total cross clamp time was 80 min with 120 min as the total cardiopulmonary bypass time. The patient was extubated after 14 h. The postoperative course was uneventful, and the patient was discharged on the 12th postoperative day including five days in cardiac surgery department, and seven days in satellite hospital for following up. A postoperative enhanced CT of the chest was performed one-month post-CABG and the coronary reconstructions by GSV revealed patent (Fig. 3). The histopathological results of the two aneurysms showed the degeneration due to atherosclerosis (Fig. 4).

## 3 Discussion

Aneurysmal coronary artery disease includes coronary artery ectasia (CAE) and coronary artery aneurysms (CAA). Until now, there is confusion regarding the usage of these two terms because of the lack of uniformity in the classification criteria [11]. Both terms are used to describe the excessive dilatation of the coronary arteries by 1.5 times in diameter in comparison to adjacent coronary arteries. However, the term “*aneurysms*” is used for localized dilatation whereas the term “*ectasia*” is used for representing diffuse dilatation involving more than 50% of the total length of the coronary artery [1,12,13]. In our case, the total length of the dilatation accounted for more than 50% of the length of the right coronary artery. That was a reason why we used the term “*ectasia*” instead of “*aneurysm*” for this case. Based on morphology, Markis et al. proposed a classification of CAE comprising of four types: type I (diffuse dilatation in more than two coronary vessels), type II (diffuse ectasia in a vessel associated with localized dilatation in the other vessel), type III (diffuse ectasia located in only one vessel), type IV (localized or segmental involvement) [14]. According to this morphological classification, our case was a type III CAE. Based on the size of dilatation, CAE is classified into 3 levels depending on maximal diameter: small (< 5 mm), medium (5–8 mm), and giant (>8 mm) [15]. In our case, the maximal diameter of two aneurysmal sacs were 36 mm and 43 mm, respectively. This size was more than around 5 folds compared to the criteria of a “giant” CAE. Giant size is a rare condition with only sporadic reports in the global literature.

A small aneurysm or ectasia can be asymptomatic and incidentally detected on coronary angiography. However, a giant aneurysmal sac with thrombus can be symptomatic and lead to a wide range of detrimental and life-threatening complications such as: (1) the thrombus can move and block the distal coronary arteries causing myocardial ischemia or infarction, (2) the giant mass can compress the adjacent structures, (3) the rupture of aneurysmal mass can cause cardiac tamponade or pericardial thrombus [[6], [7], [8], [9]]. Therefore, early diagnosis and treatment are crucial to saving patients. Until now, however, there is no consensus on optimal strategy for the management of CAE and CAA due to lack of controlled trials or official guidelines. Some possible treatment modalities presented in global literature include medical therapy (antiplatelet or anticoagulation therapy), coronary intervention (covered stent or coil embolization), or surgical management [15,16]. The optimal strategy should be individualized based on the size, morphology of aneurysms and associated lesions of each patient. For giant CAE and CAA, surgical management is suggested and associated with a favourable outcome. In our case, for instance, the procedure was smoothly performed with resection of two aneurysms and concomitant CABG.

Atherosclerosis is considered as the common etiologic factor responsible for CAE in adults whereas Kawasaki disease is the common cause in children and young adults [5]. In our case, although patient is a young adult, the histopathological results of the two aneurysms showed the degeneration due to atherosclerosis. This is a rare condition. However, review of the global literature suggests that atherosclerosis is present in young adults and many necropsy studies have demonstrated that changes in the vessel wall begin early in life [17,18]. Primary prevention of this disease must begin in childhood or adolescence [19].

## 4 Conclusions

Giant CAE is a rare clinical condition and is very dangerous for patients unless properly treated. Aneurysmal resection and concomitant coronary artery bypass surgery is the proper strategy for the treatment of giant aneurysmal sacs. We successfully treated a case of giant right coronary artery ectasia with favorable outcome.

## 5 Contributors

Tran Quyet Tien did operation and wrote this case report.

## Funding

No specific grant from funding agencies in the public, commercial, or not-for profit sectors supported the publication of this case report.

## Consent

Written informed consent was obtained from the patient for publication of this report and accompanying images. A copy of the written consent is available for review by the Editor-in-Chief of this journal on request.

## Consent

Written informed consent was obtained from the patient for the publication of this case report.

## Funding

No funding was received for this case report.

## Ethical approval

My Institute’s (Cardiovascular Center) representative was fully aware of this submission and this scientific activity including writing manuscript was approved by the Ethic Committee of Cho Ray hospital, where the patients were operated.

## Research studies

This work does not apply as it is a case report of a patient who has given written consent and has been de-identified. It is therefore not prospective research involving human participant.

## Guarantor

Associate Professor Tran Quyet Tien, MD, PhD - Head of Department of Cardiovascular and Thoracic Surgery, Faculty of Medicine, University of Medicine and Pharmacy at Ho Chi Minh City; Director of Cardiovascular Center of Cho Ray Hospital, Ho Chi Minh city.

## Provenance and peer review

Not commissioned, externally peer-reviewed.

## Declaration of Competing Interest

The author declares that they have no competing interests.
